# STIM1L traps and gates Orai1 channels without remodeling the cortical ER

**DOI:** 10.1242/jcs.164228

**Published:** 2015-04-15

**Authors:** Sophie Saüc, Monica Bulla, Paula Nunes, Lelio Orci, Anna Marchetti, Fabrice Antigny, Laurent Bernheim, Pierre Cosson, Maud Frieden, Nicolas Demaurex

**Affiliations:** 1 Department of Cell Physiology and Metabolism, University of Geneva, 1 Rue Michel-Servet CH-1211, Geneva 4, Switzerland; 2 Basic Neurosciences, University of Geneva, 1 Rue Michel-Servet CH-1211, Geneva 4, Switzerland

**Keywords:** Calcium signaling, Cell signaling, Electron microscopy, Ion channels, Muscle physiology

## Abstract

STIM proteins populate and expand cortical endoplasmic reticulum (ER) sheets to mediate store-operated Ca^2+^ entry (SOCE) by trapping and gating Orai channels in ER-plasma membrane clusters. A longer splice variant, STIM1L, forms permanent ER-plasma membrane clusters and mediates rapid Ca^2+^ influx in muscle. Here, we used electron microscopy, total internal reflection fluorescence (TIRF) microscopy and Ca^2+^ imaging to establish the trafficking and signaling properties of the two STIM1 isoforms in *Stim1^−/−^/Stim2^−/−^* fibroblasts. Unlike STIM1, STIM1L was poorly recruited into ER-plasma membrane clusters and did not mediate store-dependent expansion of cortical ER cisternae. Removal of the STIM1 lysine-rich tail prevented store-dependent cluster enlargement, whereas inhibition of cytosolic Ca^2+^ elevations or removal of the STIM1L actin-binding domain had no impact on cluster expansion. Finally, STIM1L restored robust but not accelerated SOCE and clustered with Orai1 channels more slowly than STIM1 following store depletion. These results indicate that STIM1L does not mediate rapid SOCE but can trap and gate Orai1 channels efficiently without remodeling cortical ER cisternae. The ability of STIM proteins to induce cortical ER formation is dispensable for SOCE and requires the lysine-rich tail of STIM1 involved in binding to phosphoinositides.

## INTRODUCTION

Store-operated Ca^2+^ entry (SOCE) is an evolutionarily conserved signaling mechanism induced by the Ca^2+^ depletion of the endoplasmic reticulum (ER) that sustains long-lasting cytosolic Ca^2+^ signals required for transcription, cell proliferation and effector function (Hogan et al., 2010; Parekh, 2010). SOCE is mediated by the ER Ca^2+^ sensors stromal interaction molecules (STIMs) STIM1 and STIM2 (Liou et al., 2005; Roos et al., 2005; Zhang et al., 2005) and the plasma membrane Ca^2+^-permeable channels Orai1, Orai2 and Orai3 (Feske et al., 2006; Vig et al., 2006; Zhang et al., 2006). STIM1 is a single-pass transmembrane ER protein bearing a luminal Ca^2+^-binding EF-hand domain (Liou et al., 2005; Stathopulos et al., 2008; Zhang et al., 2005) and a cytosolic channel activation domain (CAD) that mediates the trapping and gating of Orai channels (Kawasaki et al., 2009; Park et al., 2009; Yuan et al., 2009). Orai channels are four-transmembrane-domain proteins that assemble as tetramers (Demuro et al., 2011; Ji et al., 2008; Madl et al., 2010; Mignen et al., 2008; Penna et al., 2008) or hexamers (Hou et al., 2012) to form Ca^2+^-conducting channels in the plasma membrane (Prakriya et al., 2006; Vig et al., 2006; Yeromin et al., 2006). Upon ER Ca^2+^ depletion, Ca^2+^ dissociation from the STIM1 EF-hand domain initiates the multimerization of STIM dimers into higher-order oligomers (Liou et al., 2005; Stathopulos et al., 2006) and induces conformational changes in the cytosolic domains that release the lysine-rich tail of STIM1 and expose the CAD (Covington et al., 2010; Korzeniowski et al., 2009; Luik et al., 2008; Muik et al., 2011; Zhang et al., 2005). This favors STIM1 translocation to the plasma membrane and the formation of STIM–Orai clusters at ER-plasma membrane junctions (Luik et al., 2006; Xu et al., 2006), where interactions between CAD and Orai1 N- and C-termini (Derler et al., 2013; Park et al., 2009; Zhou et al., 2010) promote pore opening and localized Ca^2+^ influx (Luik et al., 2006; Xu et al., 2006). The steps linking store depletion to channel opening involve STIM1 multimerization, plasma membrane translocation, and co-clustering with Orai1, a process that typically takes 1–2 minutes to complete (Lewis, 2011; Sampieri et al., 2009; Wu et al., 2006). SOCE activation is associated with extensive remodeling of the ER (Shen et al., 2011), leading to the formation of characteristic structures appearing on the electron microscope as thin, elongated ER cisternae deprived of ribosomes located in close proximity (8–10 nm) to the plasma membrane (Lur et al., 2009; Orci et al., 2009; Wu et al., 2006). These cortical ER cisternae, also known as junctional ER, remain connected with the bulk ER and increase both in number and length upon ER Ca^2+^ depletion or STIM1 overexpression.

STIM1L is a longer isoform of STIM1 that is generated by alternative splicing of the *STIM1* gene, expressed predominantly in skeletal muscle and brain in rodents (Darbellay et al., 2011) and in muscle in humans (Horinouchi et al., 2012). STIM1L contains 106 additional amino acids bearing an actin-binding domain (ABD) that anchors STIM1L to the actin cytoskeleton, favoring its pre-clustering together with Orai1 at ER-plasma membrane contact sites before store depletion (Darbellay et al., 2011). STIM1L–Orai1 pre-clustering is thought to participate in the rapid activation of SOCE observed in skeletal muscle (Edwards et al., 2010), which sustains store refilling during high-frequency stimulations (Darbellay et al., 2011). The importance of SOCE for muscle cell function is highlighted by the muscular defects associated with STIM1 and Orai1 deficiencies in mice and humans (Feske, 2009). Patients with inactivating mutations in either STIM1 (e.g. E136X) or Orai1 (e.g. R91W) suffer from congenital myopathy with global muscular hypotonia (Feske, 2009) and *Stim1*-deficient mice exhibit a severe myopathy associated with perinatal mortality and with myotubes that rapidly fatigue during repeated stimulation (Stiber et al., 2008). STIM1 and Orai1 colocalize at the triad in mice and SOCE is severely blunted in flexor digitoris brevis fibers from adult transgenic mice expressing a muscle-specific dominant-negative Orai1 (E108Q) (Wei-LaPierre et al., 2013). Furthermore, gain-of-function mutations in STIM1 and Orai1 were recently associated with myopathy with tubular aggregates in two cohorts of patients (Böhm et al., 2013; Nesin et al., 2014). The deleterious effect of constitutively active SOCE in these patients appears to be specific for skeletal muscle, suggesting that muscle cells are particularly sensitive to dysregulation in STIM1–Orai1 coupling. Whether this increased sensitivity reflects differences in the ability of STIM1L to trap and gate Orai1 channels at ER-plasma membrane junctions is not known however.

To establish the intrinsic properties of the STIM1L isoform, we independently re-expressed STIM1 and STIM1L in murine embryonic fibroblasts (MEFs) ablated for both *Stim1* and *Stim2* genes (DKO cells). These cells provide a clean genetic background enabling us to ascribe unambiguously a phenotype to the expressed protein. Using Ca^2+^ imaging, total internal reflection fluorescence (TIRF) microscopy and electron microscopy, we observed that, contrary to STIM1, STIM1L mediates robust SOCE without remodeling cortical ER cisternae. In addition, Orai1 did not colocalize with STIM1L prior to store depletion and was recruited more slowly to plasma membrane clusters by STIM1L than by STIM1.

## RESULTS

### STIM1L is poorly recruited to the plasma membrane upon store depletion yet mediates robust SOCE

**Fig. 1.**
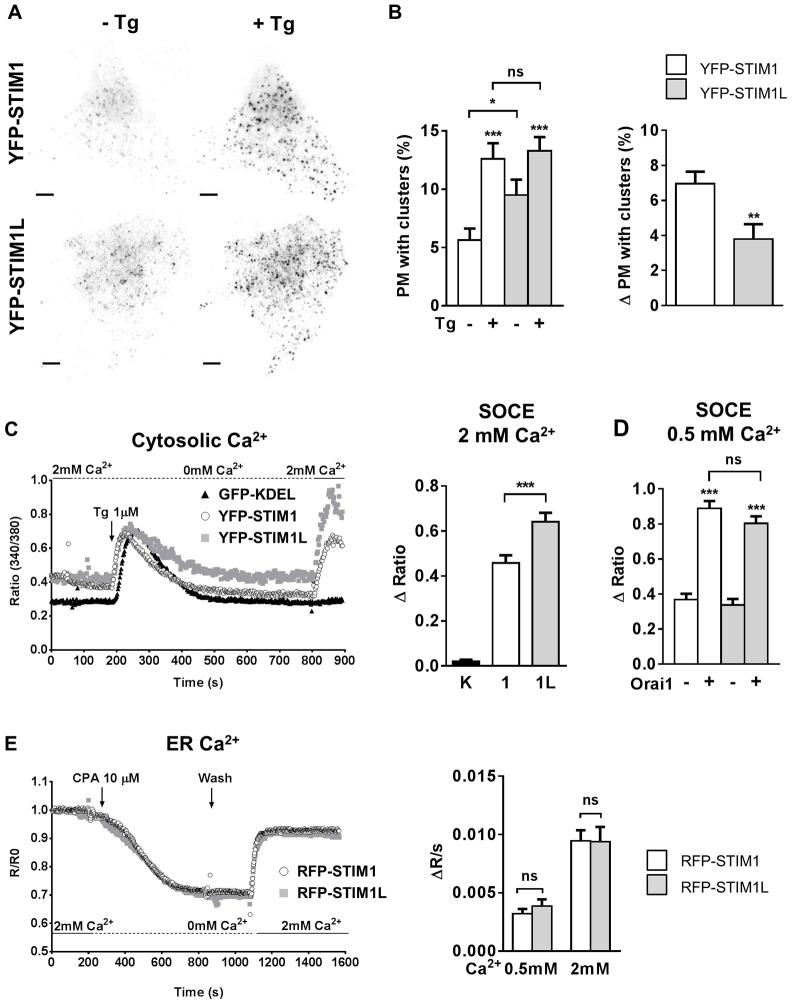
**STIM1L is poorly recruited to the plasma membrane upon store depletion yet mediates robust SOCE.**(A) TIRF images of DKO MEFs expressing YFP–STIM1 and YFP–STIM1L before (left) and 10 min after addition of 1 µM thapsigargin (Tg) (right). Scale bars: 5 µm. (B) Left, percentage of plasma membrane (PM) decorated by fluorescent clusters before and after the addition of thapsigargin. Right, percentage of plasma membrane decorated by new clusters after thapsigargin addition (*n* = 20/7/3 and 25/7/3 cells/recordings/transfections for YFP–STIM1 and YFP–STIM1L, respectively). (C) Ca^2+^ elevations evoked by the re-addition of 2 mM Ca^2+^ to cells treated with thapsigargin for 10 min in Ca^2+^-free medium (left), and quantification of thapsigargin-induced Ca^2+^ influx in 2 mM Ca^2+^ [right, *n* = 14/2/2, 35/9/6 and 51/9/6 cells/recordings/transfections for GFP–KDEL (K), YFP–STIM1 (1) or YFP–STIM1L (1L) cells, respectively]. (D) Quantification of thapsigargin-induced Ca^2+^ influx in 0.5 mM Ca^2+^ in cells expressing YFP–STIM1 (white bars) or YFP–STIM1L (gray bars) alone (*n* = 44/13/4 and *n* = 57/15/4, respectively) or together with Orai1 (*n* = 37/10/3 and *n* = 53/11/3 cells/recordings/transfections, respectively). (E) Representative changes in D1_ER_ ratio fluorescence measured in DKO cells co-transfected with D1_ER_ and either RFP–STIM1 or RFP–STIM1L. Cells were treated with 10 µM CPA for 10 min in Ca^2+^-free medium to induce store depletion, then CPA was removed by exchanging the bath solution, and 2 mM Ca^2+^ was added 3 min later to promote store refilling (left). Right, statistical evaluation of ER refilling velocity upon 0.5 mM or 2 mM Ca^2+^ re-addition (*n* = 25/11/3, 25/12/3 cells/recordings/transfections at 0.5 mM and 17/7/3, 17/9/3 cells/recordings/transfections at 2 mM for RFP–STIM1 or RFP–STIM1L cells, respectively). Data show the mean±s.e.m.; *P<0.05; **P<0.01; ***P<0.001; ns, not significant (paired Student's *t*-test for B, left, unpaired Student's *t*-test for other data).

To assess whether the additional 106 amino acids of STIM1L and its actin-binding domain conferred specific trafficking and functional properties to the long isoform, we independently re-expressed the STIM1 and STIM1L isoforms in DKO cells. We first determined the ability of the isoforms to reach the plasma membrane and form clusters following Ca^2+^ depletion of the ER by TIRF imaging, using DKO cells expressing similar levels of YFP–STIM1 or YFP–STIM1L as assessed by wide-field fluorescence imaging (supplementary material Fig. S1). A diffuse staining with few discrete fluorescence clusters was observed at rest, and new clusters appeared in the TIRF plane following passive store depletion with the sarco-endoplasmic reticulum Ca^2+^ ATPase (SERCA) inhibitor thapsigargin in both STIM1- and STIM1L-expressing cells (Fig. 1A). Quantitative analysis of the TIRF images revealed that, at rest, a smaller percentage of plasma membrane was decorated by fluorescent YFP–STIM1 clusters than YFP–STIM1L clusters (5.6% versus 9.4%, Fig. 1B, left panel). The addition of thapsigargin increased plasma membrane coverage to similar values (12.5% and 13.2%), the new YFP–STIM1 clusters recruited following thapsigargin treatment covering 6.9% of the plasma membrane versus 3.8% in YFP–STIM1L cells (Fig. 1B, right panel).

The reduced effect of thapsigargin in promoting STIM1L plasma membrane recruitment suggested that STIM1L could mediate SOCE less efficiently upon store depletion. We therefore performed Ca^2+^ imaging experiments to determine the magnitude of SOCE in DKO cells expressing either a control ER-targeted fluorescent protein (GFP–KDEL) or the two tagged STIM1 isoforms. Basal Ca^2+^ levels were increased by STIM1 re-expression, more markedly in YFP–STIM1 cells which, unlike YFP–STIM1L cells, were more sensitive to Ca^2+^ removal, consistent with increased basal Ca^2+^ influx (Fig. 1C; supplementary material Fig. S1). As expected, robust Ca^2+^ elevations were observed upon re-addition of 2 mM Ca^2+^ to thapsigargin-treated YFP–STIM1 or YFP–STIM1L cells, whereas essentially no response was observed in cells expressing GFP–KDEL (Fig. 1C). Unexpectedly, the amplitude of the thapsigargin-induced Ca^2+^ elevations was higher in YFP–STIM1L cells than in YFP–STIM1 cells, suggesting that the long isoform is more potent in mediating SOCE. To verify this observation, we measured SOCE in cells co-expressing Orai1 channels and those without Orai1 co-expression, using a lower Ca^2+^ concentration (0.5 mM) to avoid fura-2 saturation during Ca^2+^ re-addition. Under these conditions, no differences were observed between YFP–STIM1 or YFP–STIM1L cells regardless of Orai1 co-expression (Fig. 1D). To check whether the reduced plasma membrane recruitment of STIM1L could cause less efficient ER refilling, we measured the changes in ER Ca^2+^ concentration in cells transiently exposed to the reversible SERCA inhibitor cyclopiazonic acid (CPA, Fig. 1E). ER refilling proceeded with similar kinetics in cells expressing RFP–STIM1 or RFP–STIM1L upon re-addition of either 0.5 mM or 2 mM Ca^2+^ (Fig. 1E, right panel), whereas no response was observed in GFP–KDEL cells (supplementary material Fig. S1). These data indicate that, despite its reduced ability to translocate to the plasma membrane in response to thapsigargin, STIM1L mediates SOCE and ER refilling at least as efficiently as the classical isoform.

### STIM1L does not enlarge plasma membrane clusters upon store depletion

**Fig. 2.**
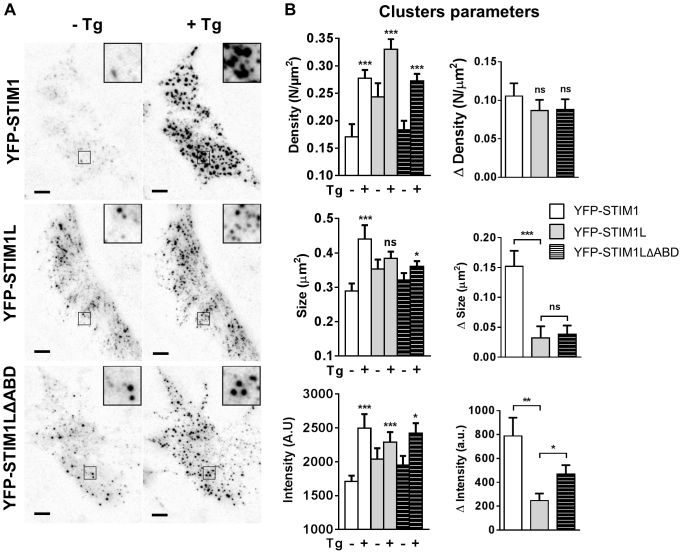
**STIM1L does not enlarge plasma membrane clusters upon store depletion.**(A) TIRF images of cells expressing YFP–STIM1, YFP–STIM1L and STIM1LΔABD taken before (left) and 10 min after thapsigargin (Tg) addition (right). Insets, a threefold magnification to show the morphology of fluorescent clusters. Scale bars: 5 µm. (B) Quantitative analysis of TIRF images showing the effects of thapsigargin on the density, size and intensity of YFP clusters. Right panels show the absolute increase in cluster density, size and intensity following thapsigargin addition (*n* = 20/7/3, 25/7/3 and 34/7/3 cells/recordings/transfections for YFP–STIM1, YFP–STIM1L and STIM1LΔABD. A.U., arbitrary units. Data show the mean±s.e.m.; *P<0.05; **P<0.01; ***P<0.001; ns, not significant [paired Student's *t*-test (left), unpaired Student's *t*-test (right)].

Careful examination of the plasma membrane clusters forming following store depletion revealed that YFP–STIM1-containing clusters were larger and denser than YFP–STIM1L clusters (Fig. 2A). Morphometric analysis indicated that ER Ca^2+^ depletion induced the appearance of a similar amount of new plasma membrane clusters in cells expressing the long and short isoform (Fig. 2B, top panels), and confirmed that YFP–STIM1 clusters increased in size and intensity upon ER depletion, by 53% and 46%, respectively (Fig. 2B, white bars). In contrast, the size of YFP–STIM1L clusters did not increase significantly following thapsigargin addition and their mean intensity increased only by 12% (Fig. 2B, gray bars). These data indicate that, unlike STIM1, STIM1L is unable to form large plasma membrane clusters upon ER Ca^2+^ depletion and is recruited less efficiently upon thapsigargin addition. In myotubes, STIM1L interacts with the actin cytoskeleton and deletion of ten amino acids (589–599) within the additional domain hinders this actin tethering (Darbellay et al., 2011). To investigate whether the reduced efficiency of STIM1L in forming large and bright plasma membrane clusters was due to actin binding, we tested the effects of a mutant lacking the actin-binding domain (STIM1LΔABD). Expression of the STIM1LΔABD mutant was as efficient as that of the native isoform in rescuing SOCE (supplementary material Fig. S2), indicating that actin binding does not interfere with STIM1L-mediated channel opening. Disruption of the actin-binding domain had no significant effect on the number of new YFP–STIM1L clusters forming upon store depletion or on the mean cluster size, but partially restored the evoked increase in fluorescence intensity (Fig. 2B, hatched bars). These data indicate that actin binding hinders the ability of STIM1L to populate existing clusters upon store depletion but does not account for its inability to enlarge plasma membrane clusters.

### STIM1L does not recruit and enlarge cortical ER cisternae upon store depletion

**Fig. 3.**
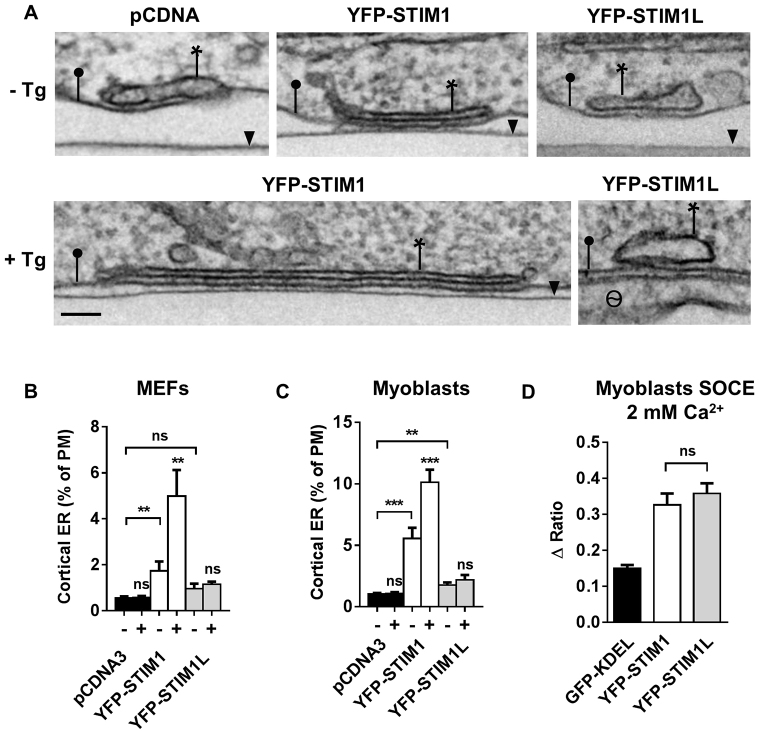
**STIM1L does not recruit cortical ER cisternae upon store depletion.**(A) Ultra-structural analysis of DKO cells expressing pCDNA, YFP–STIM1 or YFP–STIM1L before (top) or 10 min after exposure to 1 µM thapsigargin (Tg) (bottom). Images show sheets of cER (asterisks) apposed to the plasma membrane (closed circle). Arrowhead denotes the dish bottom; θ marks an adjacent cell. Scale bar: 100 nm. (B,C) Percentage of plasma membrane (PM) decorated by cER in DKO cells (B) and myoblasts (C) before and after addition of thapsigargin (*n* = 71–102 for each condition from at least three independent experiments). (D) Quantification of the Ca^2+^ elevations evoked by Ca^2+^ re-addition to thapsigargin-treated myoblasts expressing GFP–KDEL, YFP–STIM1 or YFP–STIM1L (*n* = 25/5/3, 27/5/3 and 26/5/3 cells/recordings/transfections, respectively) Quantitative data show the mean±s.e.m.; **P<0.01; ***P<0.001; ns, not significant (unpaired Student's *t*-test).

To better characterize the plasma-membrane-associated structures populated by the two STIM1 isoforms, we quantified the formation of ER-plasma membrane contacts by electron microscopy. Earlier studies in HeLa cells indicated that STIM1 overexpression per se increases the amount of cortical ER (cER) and that the frequency and the size of these structures is further increased by ER Ca^2+^ depletion (Orci et al., 2009; Wu et al., 2006). To allow a quantitative comparison, populations expressing similar levels of YFP–STIM1 or YFP–STIM1L were selected by fluorescence-activated cell sorting (FACS) (supplementary material Fig. S3). Consistent with earlier findings in HeLa cells, STIM1 expression induced the formation of cER in resting DKO cells and thapsigargin promoted the appearance of characteristic long and thin cER sheets (Fig. 3A). Quantification of the electron microscopy images indicated that STIM1 expression increased the percentage of plasma membrane bearing apposed ER cisternae by threefold and that thapsigargin stimulation further increased this value by the same factor, such that ∼5% of the plasma membrane was eventually tethered to cER (Fig. 3B). In stark contrast, expression of STIM1L caused a mild increase in the amount of cER (+73%, *P* = 0.04), and this value did not increase further upon thapsigargin stimulation (Fig. 3B). Qualitative differences were also evident, as we failed to detect long and thin cER sheets in YFP–STIM1L cells treated with thapsigargin (Fig. 3A). We conclude that compared to STIM1, STIM1L is characterized by a low ability to induce cER when expressed, and does not induce further ER remodeling upon thapsigargin stimulation, as suggested by the TIRF experiments reported above. To verify that the failure of STIM1L to remodel the ER was not due to an adaptation of the DKO cells permanently depleted of both STIM1 and STIM2, we repeated the ultra-structural and functional measurements in myoblasts, which express STIM1 but not STIM1L, before their differentiation into myotubes (Darbellay et al., 2011). Expression of STIM1 induced robust cER formation in resting cells (+540%), and stimulation with thapsigargin further increased the amount of cER by twofold (Fig. 3C). Expression of STIM1L caused a significant but limited increase in cER (+71%), to levels that did not further increase upon thapsigargin stimulation (Fig. 3C). Store-dependent cER expansion mediated by STIM1 also occurred at physiological STIM1 concentrations, as thapsigargin increased the amount of cER by ∼65% in naïve HeLa cells (supplementary material Fig. S3). Thus, compared to STIM1, STIM1L is characterized by a low ability to induce cER and an undetectable response to thapsigargin stimulation. STIM1L and STIM1 induced similar Ca^2+^ fluxes when expressed in human myoblasts (Fig. 3D), indicating that the two isoforms can gate SOCE channels in muscle.

### STIM1L mediates slowly activating SOCE and delayed Orai1 clustering

**Fig. 4.**
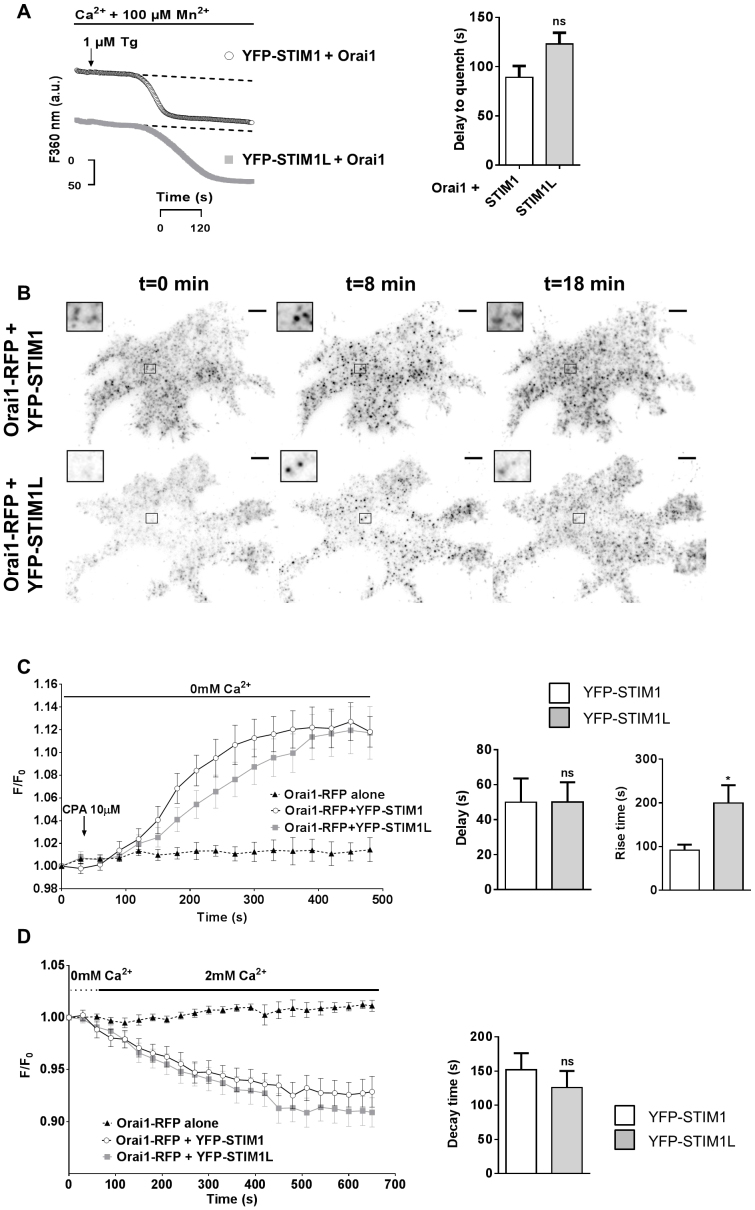
**STIM1L mediates slowly activating SOCE and delayed Orai1 clustering.**(A) Representative Mn^2+^ quench recordings (left) and quantification (right) of DKO cells co-transfected with Orai1 together with YFP–STIM1 or YFP–STIM1L (*n* = 20/6/2 and 23/7/2 cells/recordings/transfections, respectively). Cells were exposed to 100 µM Mn^2+^ prior to thapsigargin (Tg) addition, and fura-2 fluorescence quench was measured at 360 nm. a.u., arbitrary units. (B) TIRF images of DKO cells expressing Orai1–RFP together with YFP–STIM1 or YFP–STIM1L taken before (left), 8 min after addition of the reversible SERCA inhibitor CPA (middle) and 10 min after CPA removal and Ca^2+^ re-addition (right). Insets show a threefold magnification of Orai1–RFP clusters. Scale bars: 5 µm. (C) Changes in Orai1–RFP TIRF fluorescence evoked by store depletion in cells expressing Orai1–RFP alone or together with YFP–STIM1 or YFP–STIM1L (*n* = 6, 11 and 13, respectively). CPA (10 µM) was added at *t* = 35 s. Right panels show the delay between CPA addition and fluorescence increase, and the time to reach half-maximal fluorescence. (D) Changes in Orai1–RFP TIRF fluorescence evoked by subsequent store refilling in the cells shown in C. CPA was removed by exchanging the bath solution and 2 mM Ca^2+^ was added at *t* = 35 s. Right, fluorescence decay time. Quantitative data show the mean±s.e.m.; *P<0.05; ns, not significant (unpaired Student's *t*-test).

STIM1L was shown previously to drive rapid SOCE influx when exogenously expressed and to colocalize with Orai1–RFP plasma membrane clusters in resting myoblasts (Darbellay et al., 2011). To test whether these properties are intrinsic to the STIM1L protein, we measured the kinetics of SOCE activation with the Mn^2+^ quench technique in DKO cells expressing either STIM1 or STIM1L. Cells were transfected with Orai1 together with the YFP-tagged STIM1 isoform, incubated with 100 µM Mn^2+^, a Ca^2+^ surrogate that quenches the fluorescence of fura-2, and treated either with ATP+thapsigargin to induce fast ER Ca^2+^ depletion or with thapsigargin alone to slowly deplete the ER. A decrease in fura-2 fluorescence was observed in both STIM1- and STIM1L-expressing cells after a similar 25-s delay for fast depletion (supplementary material Fig. S4) and 2-min delay for slow depletion (Fig. 4A). To test whether this delay reflected the time required for the trapping of SOCE channels by STIM1 isoforms, we then measured the changes in Orai1–RFP plasma membrane fluorescence by TIRF imaging upon slow Ca^2+^ depletion. A diffuse plasma membrane fluorescence pattern with discrete fluorescence puncta was observed in cells transfected with Orai1–RFP alone (not shown). This resting pattern was not perturbed by the co-expression of either YFP–STIM1 or YFP–STIM1L (Fig. 4B). Store depletion had no effect in cells transfected with Orai1–RFP alone and induced the formation of Orai1–RFP clusters in cells co-expressing either YFP–STIM1 or YFP–STIM1L (Fig. 4B). Time-lapse imaging revealed that Orai1–RFP clusters formed more slowly in cells expressing YFP–STIM1L compared to YFP–STIM1 (Fig. 4C). The delay between the addition of the reversible SERCA inhibitor CPA and the initiation of cluster formation was identical, but the kinetics of the subsequent fluorescence increase, reflecting the accumulation of Orai1 proteins in STIM1 plasma membrane clusters, was ∼2 min slower in YFP–STIM1L expressing cells (Fig. 4C). This indicates that, unlike in myoblasts, STIM1L is not bound to Orai1 in resting DKO cells and captures Orai1 channels more slowly than STIM1 upon store depletion. We then reasoned that, despite being unable to immobilize Orai1 channels at rest, the STIM1L isoform might retain the incoming channels after their binding and activation in plasma membrane clusters. We thus followed the kinetics of Orai1–RFP plasma membrane cluster dissociation by exposing cells to CPA and then re-adding Ca^2+^ to refill intracellular stores. Ca^2+^ re-addition restored resting [Ca^2+^]_ER_ levels (Fig. 1E) and evoked a rapid decrease in the Orai1–RFP plasma membrane fluorescence, indicative of de-clustering (Fig. 4D, left). The Orai1–RFP clusters dissociated with identical kinetics in cells expressing the short or the long isoform (Fig. 4D, right). These data indicate that exogenous STIM1L expression does not intrinsically confer a kinetic advantage in the activation of SOCE, as reported previously for myoblasts, and that the long isoform does not irreversibly trap Orai1 channels in plasma membrane clusters after their activation. Instead, the long splice variant recruits Orai1 channels more slowly than the ubiquitous isoform.

### Cluster expansion requires the STIM1 lysine-rich tail but not cytosolic Ca^2+^ elevations

**Fig. 5.**
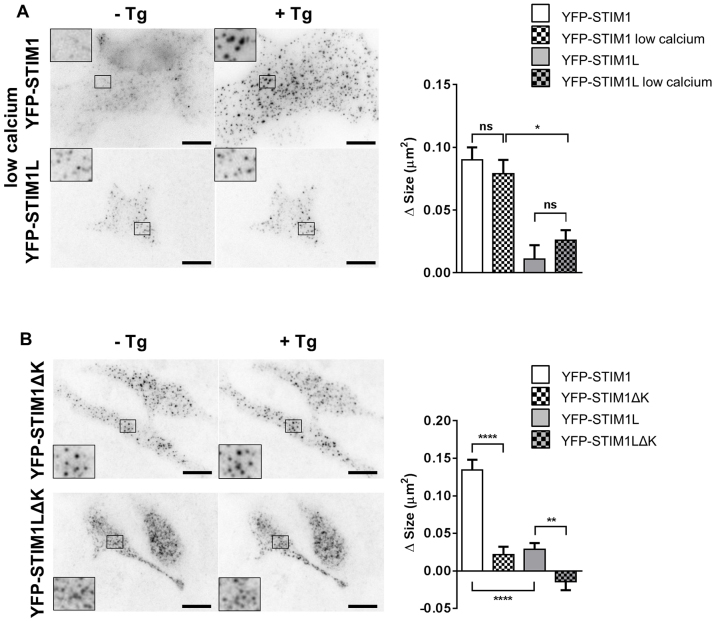
**Cluster expansion requires the STIM1 lysine-rich tail but not cytosolic Ca^2+^ elevations.**(A) Left, TIRF images of cells expressing YFP–STIM1 and YFP–STIM1L taken before (left) and 10 min after thapsigargin (Tg) addition (right) in conditions preventing cytosolic Ca^2+^ elevations (20 µM BAPTA-AM and 50 µM La^3+^). Insets, a 2.6-fold magnification to show the morphology of fluorescent clusters. Scale bars: 10 µm. Right, quantitative analysis of TIRF images showing the effects of thapsigargin on the absolute increase in cluster size. (*n* = 33/5/3, 38/5/3, 36/5/3 and 44/5/3 cells/recordings/transfections). (B) Left, TIRF images of cells expressing YFP–STIM1ΔK and YFP–STIM1LΔK taken before (left) and 10 min after thapsigargin addition (right). Right, quantitative analysis of TIRF images showing the effects of thapsigargin on the absolute increase in cluster size. (*n* = 41/7/6, 49/6/5, 51/7/5 and 42/6/5 cells/recordings/transfections). Quantitative data show the mean±s.e.m.; *P<0.05, **P<0.01; ****P<0.0001; ns, not significant (unpaired Student's *t*-test).

To gain insight into the mechanism underlying the different remodeling capacities of the STIM1 isoforms, we tested whether cytosolic Ca^2+^ elevations are required for cluster enlargement, using a combination of BAPTA-AM to clamp the cytosolic Ca^2+^ concentration at nanomolar levels and La^3+^ to prevent Ca^2+^ entry. Under these Ca^2+^-clamped conditions, store depletion enlarged YFP–STIM1 clusters as effectively as under control conditions, whereas YFP–STIM1L clusters remained poorly responsive (Fig. 5A). This indicates that the different abilities of the two isoforms to enlarge clusters are not due to differences in the subplasmalemmal Ca^2+^ concentration. Next, we tested whether the different abilities of the two STIM1 isoforms to enlarge plasma membrane clusters are linked to their capacity to bind to phosphatidylinositol 4,5-bisphosphate (PIP_2_)-rich plasma membrane domains. For this purpose, we generated STIM1 mutants lacking the lysine-rich terminal cytosolic domain (YFP–STIM1ΔK), which was shown previously to mediate the trapping of STIM1 by PIP_2_ (Liou et al., 2007). The YFP–STIM1ΔK mutant accumulated in clusters that enlarged minimally following store depletion, mimicking the behavior of the full-length YFP–STIM1L (Fig. 5B). The corresponding truncation rendered STIM1L isoform clustering completely insensitive to thapsigargin (Fig. 5B). These data demonstrate that the ability of STIM1 to enlarge plasma membrane clusters requires its lysine-rich tail and, thus, likely reflects binding to phosphoinositides.

## DISCUSSION

In this study, we demonstrate that STIM1L, the long splice variant of skeletal muscle, can recruit and activate plasma membrane Orai1 channels in a store-operated manner without remodeling cortical ER cisternae. This behavior contrasts with the known ER remodeling properties of the shorter ubiquitous STIM1 protein, whose re-expression in DKO fibroblasts led to the formation of thin cortical ER sheets tethered to the plasma membrane. STIM1 expression induces cER formation in several cell types (Lur et al., 2009; Nunes et al., 2012; Orci et al., 2009; Wu et al., 2006), as do other proteins such as Ist2 and extended synaptotagmins (Ercan et al., 2009; Giordano et al., 2013). We now show that the ER remodeling action of STIM1 requires the lysine-rich tail domain that mediates STIM1 trapping by phosphoinositides. This strongly suggests that the binding of STIM1 to phosphoinositides mediates cER expansion upon store depletion. Given that STIM1L contains an identical lysine-rich tail, the inability of STIM1L to expand the cER suggests that the activity of this motif is hindered in the long STIM1 isoform. This putative steric hindrance does not reflect actin tethering, as a STIM1L mutant lacking the actin-binding domain failed to mediate cER expansion.

Despite their distinct trafficking properties, the two splice variants were able to trap Orai1 channels and to mediate both SOCE activation and ER refilling. This suggests that an increase in the amount of cortical ER is not needed for maximal activation of Orai1 channels. STIM1 gating of Orai1 channels reflects the binding of the STIM1 CAD domain to the N- and C-terminus of Orai1 (Derler et al., 2013; McNally et al., 2013; Park et al., 2009; Zheng et al., 2013; Zhou et al., 2010) and maximal channel activation occurs with two STIM1 molecules bound per Orai1 subunit (Hoover and Lewis, 2011; Li et al., 2011; Scrimgeour et al., 2009). In our DKO MEFs re-expressing STIM1 or STIM1L, sufficient STIM1 is likely available to gate all endogenous Orai1 channels, accounting for the similar potency of the two isoforms despite the fivefold difference in cortical ER. STIM1L was, in fact, more potent than STIM1 in mediating SOCE at high Ca^2+^ concentrations, but this effect did not translate into more efficient ER refilling. The smaller Ca^2+^ elevations observed in STIM1 cells at high Ca^2+^ concentrations might reflect Ca^2+^ trapping in larger contact sites, which could promote the Ca^2+^-dependent inactivation of Orai1 channels, or inefficient CAD presentation by STIM1 molecules recruited into large clusters. Interestingly, PIP_2_ levels are reduced in STIM1–Orai1 clusters organized by septins (Sharma et al., 2013), suggesting that septins might limit cER expansion by favoring PIP_2_ depletion. PIP_2_ depletion, in turn, might promote the detachment of STIM1 from the plasma membrane and reduce its ability to bind Orai1. Regardless of the underlying mechanism, the lack of correlation between the size of ER-plasma membrane contact sites and SOCE amplitude is consistent with the report that depletion of extended synaptotagmins does not impair SOCE despite a fourfold decrease in cortical ER (Giordano et al., 2013). That STIM1L promotes Orai1 clustering and SOCE activation with minimal plasma membrane translocation indicates that Orai1 channel trapping into clusters does not require the synchronous translocation of STIM1L and can occur when STIM1L molecules already present in existing clusters become activated. This is compatible with a diffusion trap model in which activated STIM1 is first trapped at ER-plasma membrane junctions and subsequently captures Orai1 diffusing in the plasma membrane plane by CAD binding. This model was recently validated by single-particle tracking (Wu et al., 2014), which showed that STIM1–Orai1 binding drastically slows diffusion of both proteins.

Our study provides several new pieces of information regarding the function of STIM1 proteins. First, we show that the long splice variant is sufficient to activate SOCE channels by expressing this protein in cells lacking all endogenous STIM isoforms. Until now, STIM1L activity had only been recorded in cells expressing detectable levels of STIM1 and STIM2 and whether the long splice variant could function independently of its shorter counterparts was unclear. Second, we dissociate the Ca^2+^ signaling properties of STIM1 proteins from their ability to remodel the ER, by showing that STIM1L activates SOCE channels without increasing the amount of cortical ER structures. This demonstrates that activation of plasma membrane channels as a result of decreased ER Ca^2+^ levels can occur without *de novo* formation or alteration of ER-plasma membrane contact sites. Third, we show that the ability of STIM1 to enlarge the cER requires its lysine-rich tail involved in phosphoinositide binding, thereby linking cER expansion to membrane lipid composition. The different capacities of cells to undergo store-dependent cER expansion might therefore reflect differences in plasma membrane lipid composition. Finally, we show that STIM1L expression is not sufficient to recapitulate the rapid SOCE that was reported in skeletal myotubes (Darbellay et al., 2011). This rapid influx correlated with the presence of pre-formed STIM1–Orai1 clusters in resting muscle cells, whereas we report here that expressed STIM1L does not trap Orai1 channels into clusters prior to store depletion in fibroblasts. Instead, Orai1 clustering was the rate-limiting step for SOCE activation, and STIM1L did not retain the recruited channels into clusters following store refilling. This suggests that additional proteins promote the formation of the permanent STIM1L–Orai1 clusters that mediate rapid influx in muscle cells. We could not recapitulate rapid influx by co-expressing STIM1L with STIM1 (data not shown), indicating that the rapid influx of Ca^2+^ into muscle cells, which express STIM1 early during differentiation and both STIM1 and STIM1L at later stages, is not due to the formation of STIM1L–STIM1 heteromers. Scaffolding and regulatory proteins such as septins, CRACR2A, junctate [an isoform of ASPH (aspartate β-hydroxylase) lacking the hydroxylase domain], Golli (an alternative splice variant of the myelin basic protein MBP) and POST (also known as SLC35G1) have been shown to interact with STIM1 and/or Orai1 and to modulate their assembly and disassembly at ER-plasma membrane junctions (reviewed in Shim et al., 2015). Whether and how these proteins stabilize STIML–Orai1 interactions in skeletal muscle is not known however. Alternatively, differences in the organization of actin might account for the different behavior of STIM1L in skeletal muscle cells and fibroblasts.

Interestingly, store depletion promoted cER expansion in HeLa cells, confirming our earlier study (Orci et al., 2009), but did not induce ER remodeling in native human myoblasts. Earlier studies reported that store depletion induces ER remodeling in mouse embryonic fibroblasts (Nunes et al., 2012) but not in primary pancreatic acinar cells (Lur et al., 2009). The reduced cER remodeling capacity of myoblasts and pancreatic acinar cells might reflect lower STIM1 expression levels, reduced plasma membrane PIP_2_ content or ER architectures that impose physical constraints on the remodeling process. Because STIM1 mediates store-dependent cER remodeling when expressed in myoblasts, the limited cER remodeling capacity of native myoblasts probably reflect their low endogenous STIM1 levels rather than low PIP_2_ levels or structural ER constraints.

**Fig. 6.**
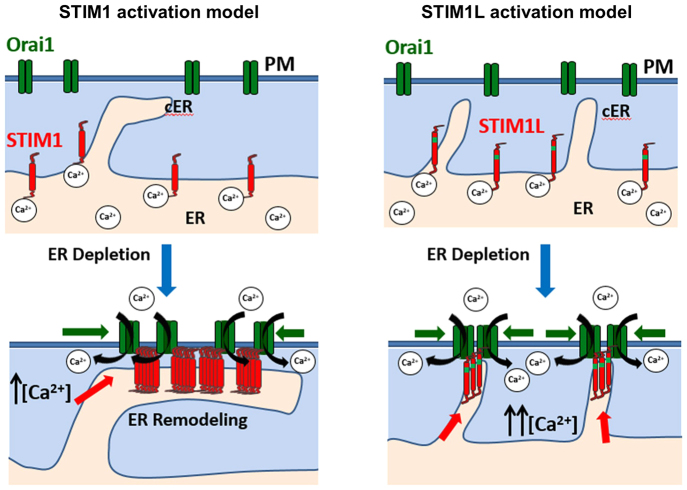
**Proposed model of STIM1 and STIM1L recruitment and activation.**(red) and STIM1L (red and green) proteins are distributed throughout the ER, and Orai1 channels (green) are dispersed on the plasma membrane (PM). Bottom, following Ca^2+^ depletion of the ER, STIM1 induces cortical ER expansion through its lysine-rich tail (red hook) and recruits Orai1 channels in large ER-plasma membrane clusters, causing submaximal SOCE activation, possibly because Ca^2+^ ions trapped in the cleft promote Orai1 channel inactivation (left). In contrast, STIM1L does not alter cortical ER structures and recruits Orai1 channels in small ER-plasma membrane clusters, causing maximal SOCE activation as entering Ca^2+^ ions readily diffuse in the cytoplasm (right).

We thus propose the following model to account for the cell signaling effects of the two STIM1 isoforms (Fig. 6). In resting cells, both STIM1 and STIM1L (red) distribute evenly throughout the ER and might populate a few discrete cortical ER structures, without interacting with Orai1 channels scattered on the plasma membrane (green). Upon ER Ca^2+^ depletion, activated STIM1 accumulates into cortical ER structures and induces their extension into large cortical ER sheets, promoting the recruitment of Orai1 channels in large plasma membrane clusters. cER expansion requires the binding of STIM to phosphoinositides through its lysine-rich tail (red hook). In contrast, STIM1L activates and/or accumulates into existing cortical ER structures without modifying their morphology, recruiting Orai1 channels in small plasma membrane clusters. Whether the ability of STIM1 to extend cortical ER structures serves other functions besides Ca^2+^ signaling is not known, but the ability of STIM1 to remodel the ER is clearly important to deliver the CAD ligand to target channels that are not located at the plasma membrane. STIM1 gates Orai1 channels on secretory granules to promote ER refilling and exocytosis (Dickson et al., 2012) and recruits ER cisternae to phagosomes to generate local Ca^2+^ elevations that boost phagocytosis (Nunes et al., 2012). In both cases, the target channels are on newly generated organelles devoid of permanent ER contact sites, and ER remodeling is required to deliver the STIM1 ligand. Conversely, the inability of STIM1L to extend the cortical ER might reflect the specialization state of muscle cells, whose fixed architecture and rhythmic sarcoplasmic reticulum Ca^2+^ release increase the requirement for plasma-membrane-attached STIM1 as gatekeeper of SOCE. STIM1L thus appears to be a specialized molecule dedicated to Ca^2+^ signaling at pre-existing contact sites, whereas STIM1 is a more versatile molecule able to remodel the ER for Ca^2+^ signaling and possibly other purposes.

## MATERIALS AND METHODS

### Materials

Thapsigargin was purchased from Sigma-Aldrich (Switzerland); cyclopiazonic acid (CPA) from Calbiochem; Fura-2/AM, Pluronic F-127 and CellMask Plasma Membrane Stain from Life Technologies (Carlsbad, CA). YFP–STIM1 was a gift from Dr Anant B. Parekh (University of Oxford, UK). YFP–STIM1L and YFP–STIM1LΔABD were constructed as described previously (Darbellay et al., 2011). RFP–STIM1 and RFP–STIM1L were created by gene synthesis, replacing YFP with RFP in both constructs (GeneCust; Dudelange, Luxembourg). YFP–STIM1ΔK was obtained from Addgene (Plasmid 18861; Cambridge, MA) and YFP–STIM1LΔK was obtained by mutagenesis (GeneCust; Dudelange, Luxembourg), Orai1 was obtained from Addgene (Plasmid 12199; Cambridge, MA) and pCMV/myc/ER/GFP (KDEL–GFP) was purchased from Life Technologies (Plasmid V823-20). D1_ER_ was kindly provided by Drs Amy Palmer and Roger Tsien (University of California, San Diego, CA; Palmer et al., 2004). Orai1–RFP was provided by Drs Dalia Al-Ansary and Barbara Niemeyer (Saarland University, Homburg, Germany; Quintana et al., 2011).

### Cell culture and transfection

*Stim1^−/−^*/*Stim2^−/−^* MEFs (DKO cells) generated by targeted gene disruption (Oh-hora et al., 2008) were a kind gift from Dr Masatsugu Oh-hora (Tokyo Medical and Dental University, Tokyo, Japan). DKO cells were maintained at 37°C in 5% CO_2_ in Dulbecco's modified eagle medium (22320-022, Life Technologies), 10% FCS, 5 µg/ml streptomycin and 5 units/ml penicillin. Cells were seeded on 25-mm diameter glass coverslips and transfected at 50% confluence with Lipofectamine 2000 (Life Technologies) by adding 2 µg of plasmid/coverslip. For TIRF imaging experiments, coverslips were coated with poly-L-lysine (Sigma). Cells were imaged 24–48 h after transfection. Muscle samples, cell dissociation and clonal culture from satellite cells were prepared as described previously (Arnaudeau et al., 2006). Human muscle samples were obtained from children without known neuromuscular disease after informed consent, as approved by the University Hospital of Geneva Research Committee on the use of humans as experimental subjects (Protocol 05-078). All work on human subjects was carried out in accordance with the Declaration of Helsinki. Myoblasts were maintained at 37°C in 5% CO_2_ in growth medium containing 15% FCS (Life Technologies), 5 µg/ml gentamicin (Gibco), 0.5 mg/ml bovine serum albumin (Sigma-Aldrich), 0.5 mg/ml fetuin (Sigma-Aldrich), 1 mM creatinine (Fluka), 0.04 mg/ml insulin, 0.39 µg/ml dexamethasone, 100 µg/ml pyruvate, 50 µg/ml uridine (Sigma-Aldrich) and 100 ng/ml epidermal growth factor (Collaborative Research). Cells were seeded on 25-mm diameter glass coverslips and transfected by electroporation with Amaxa Nucleofector II device (Lonza, Switzerland) with 2 µg of plasmid DNA.

### Ca^2+^ measurements

Changes in cytosolic Ca^2+^ concentration were measured with Fura-2. Cells were loaded with 2–4 µM Fura-2/AM plus 1 µM pluronic acid for 40 min in the dark at room temperature in a HEPES-buffered solution containing: 135 mM NaCl, 5 mM KCl, 1 mM MgCl_2_, 2 mM CaCl_2_, 10 mM HEPES, 10 mM glucose, pH adjusted at 7.45 with NaOH. Cells were washed twice and equilibrated for 10–15 min in the same buffer to allow de-esterification, before imaging on a microscope (Axio Observer, Zeiss, Germany) equipped with a Lambda DG4 illumination system (Sutter Instrument Company, Novato, CA), which rapidly changed the excitation wavelengths between 340 nm (340AF15; Omega Optical, Brattleboro, VT) and 380 nm (380AF15; Omega Optical). Emission was collected through a 415DCLP dichroic mirror and a 510WB40 filter (Omega Optical), by a cooled, 12-bit CCD camera (CoolSnap HQ, Ropper Scientific, Trenton, NJ). Experiments were performed at room temperature in HEPES-buffered solution. The Ca^2+^-free solution contained 1 mM EGTA instead of 2 mM CaCl_2_. For Mn^2+^ quench experiments, cells were excited at 360 nm (360BP10; Omega Optical). For [Ca^2+^]_ER_ measurements, cells were transiently transfected with a cameleon probe targeted to the ER (D1_ER_) and excited at 440 nm (440AF21, Omega Optical) through a 455-nm dichroic mirror (455DRLP, Omega Optical), and emission was collected alternately at 480 and 535 nm (480AF30 and 535AF26, Omega Optical) using a filter wheel (Ludl Electronic Products, Muenchen, Germany). Image acquisition and analysis were performed with Metafluor 6.3 software (Universal Imaging, West Chester, PA).

### TIRF imaging and quantification

To accurately determine the TIRF plane, the plasma membrane was labeled with CellMask Orange according to manufacturer's recommendations, and cells were bathed in Ca^2+^-containing medium. TIRF images were obtained on a Nikon Eclipse Ti microscope equipped with a Perfect Focus System (PFS III) and a 100× oil CFI Apochromat TIRF Objective (NA 1.49; Nikon Instruments Europe B.V.). For 488-nm excitation (YFP–STIM1, YFP–STIM1L and YFP–STIM1LΔABD), the filter cube contained a ZET488/10 excitation filter (Chroma Technology Corp.), a 502-nm dichroic mirror (H 488 LPXR superflat) and a 530/43 Bright Line HC emission filter (Semrock, Inc.). For 561-nm excitation (CellMask Orange, Orai1-RFP), the filter cube contained a ZET 561/10 excitation filter (Chroma Technology Corp., Bellows Falls, VT), a 580-nm dichroic mirror (H 568 LPXR superflat), and an ET605/50 emission filter (Chroma Technology Corp.). Emission signals were collected by a cooled EMCCD camera (iXon Ultra 897, Andor Technology Ltd) and images were acquired with NIS-Elements Ar software V4.13 (Nikon). All experiments were performed at room temperature (22–25°C). Expression levels of YFP-tagged STIM proteins were assessed by quantifying the wide-field fluorescence of transfected cells. In Figs 1 and 2, only cells with fluorescence intensity within the mean±s.d. were further analyzed (supplementary material Fig. S1A). In Fig. 5, where the size of the clusters was specifically analyzed, only cells having clusters of >0 µm^2^ and ≤5 µm^2^ before thapsigargin treatment were analyzed, to avoid including pre-activated cells. For these experiments, all cells presented comparable fluorescence intensity as assessed by wide-field fluorescence imaging. Quantification of YFP–STIM1, YFP–STIM1L, their respective ΔK mutants and YFP–STIM1LΔABD clusters was performed with a modified version of the Neurite Outgrowth Application Module of Metamorph software (Molecular Devices). Quantification of Orai1–RFP clustering and de-clustering was performed with ImageJ. All images were background subtracted and the mean gray intensity above a defined threshold was quantified. The threshold was determined as the mean of the gray intensity plus a standard deviation of the whole cell after 8 min of ER depletion.

### Electron microscopy

Cells expressing YFP–STIM1 and YFP–STIM1L were FACS sorted (BD FACS Vantage SE) and only cells whose fluorescence was within 10–100-fold of the background fluorescence were retained (supplementary material Fig. S3). Electron microscopy analysis was performed as described previously (Orci et al., 2009). Briefly, cells were fixed with 2% glutaraldehyde, stained with uranyl acetate, postfixed with osmium tetroxide and embedded in Epon. After sectioning, the samples were observed in a Tecnai Transmission electron microscope (FEI, Zürich, Switzerland). For quantification of the amount of cortical ER (defined as ER membrane located within 20 nm of the plasma membrane), the AnalySIS software was used.

### Statistics

Data show the mean±s.e.m. significance determined by two-tailed Student's *t*-test for paired samples in Figs 1B and 2B and unpaired samples for the other figures. *P*<0.05 was considered to be significant. Numbers in the figure legends correspond to the numbers of cells, recordings or transfections.

## Supplementary Material

Supplementary Material
