# Supplementary material for: STIM1L traps and gates Orai1 channels without remodeling the cortical ER
Source: J Cell Sci. 2015 Apr 15;128(8):1568–79. doi: 10.1242/jcs.164228 (PMC4406124; doi:10.1242/jcs.164228)
Supplement: Supplementary Material [file supp_128_8_1568__index.html]

Supplementary Material 

# STIM1L traps and gates Orai1 channels without remodeling the cortical ER

## JCS164228 Supplementary Material

**Files in this Data Supplement:**

- **Supplementary Material**
