# Supplementary material for: STIM1L traps and gates Orai1 channels without remodeling the cortical ER
Source: J Cell Sci. 2015 Apr 15;128(8):1568–79. doi: 10.1242/jcs.164228 (PMC4406124; doi:10.1242/jcs.164228)
Supplement: Supplementary Material [file supp_128.8.1568_JCS164228.pdf]

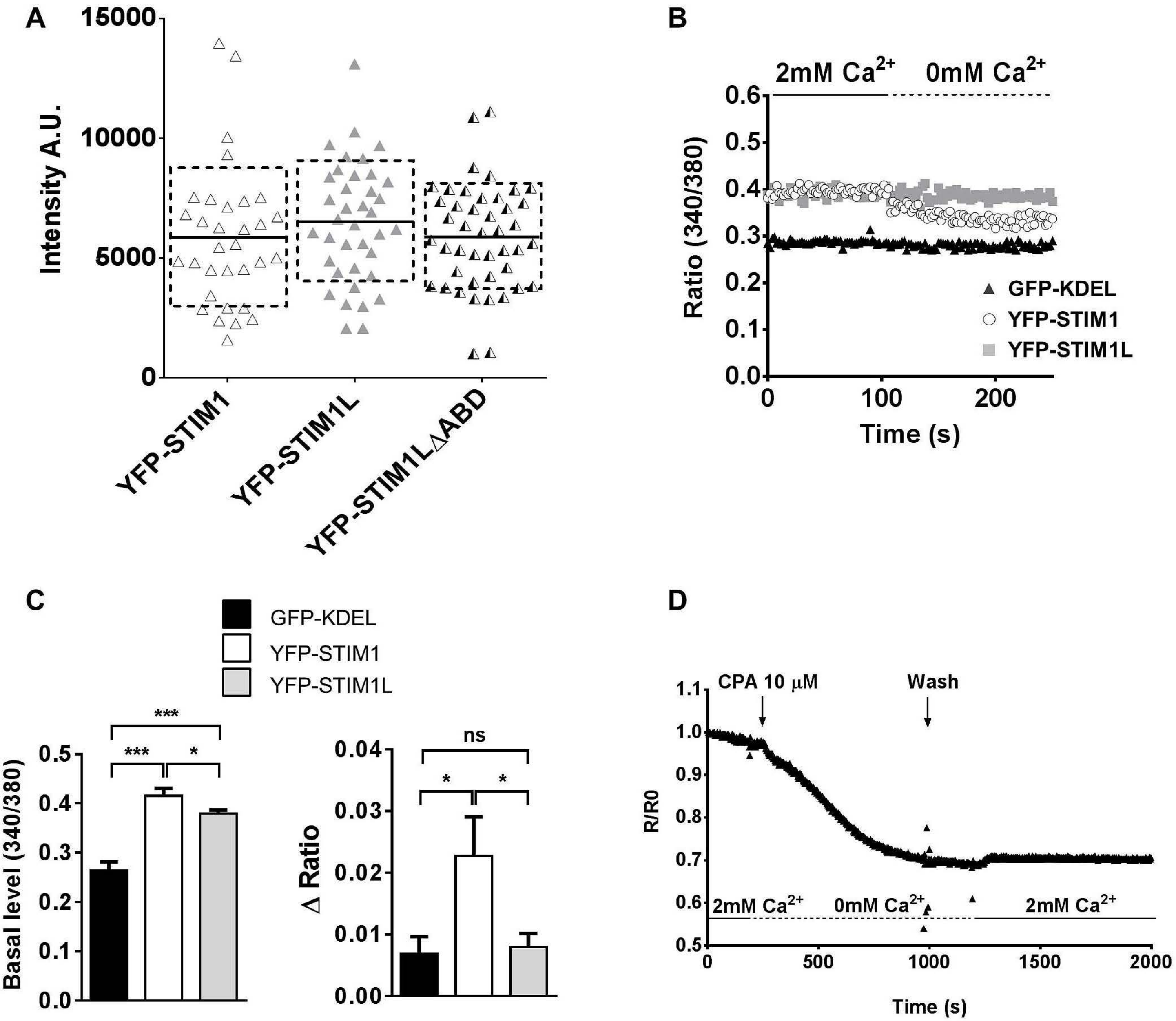

Figure S1 (related to Fig. 1). (A) Wide-field intensity of YFP-STIM1, YFP-STIM1L and YFP-STIM1LΔABD expressing cells used for TIRF imaging (n=32/8/3, 38/7/3 and 44/7/3 cells/cover-slips/experiments). Bars show the mean and dashed squares the ± 1 SD values used as criteria to select cells for quantification of TIRF images. (B) Representative Ca^{2+} recordings of DKO cells expressing KDEL-GFP, YFP-STIM1 or YFP-STIM1L during Ca^{2+} removal.(C) Statistical evaluation of basal Ca^{2+} levels (left) and of Ca^{2+} influx (right) (n=14/2/2, 35/9/6 and 51/9/6 cells/cover-slips/experiments). Basal Ca^{2+} level was evaluated as the mean ratio value before medium switch, and resting Ca^{2+} influx (DRatio) as the amplitude of the Ca^{2+} drop upon switching cells from Ca^{2+} to Ca^{2+} free medium. Unpaired t-test: *p < 0,05; **p<0,01; ***p<0,001. All errors bars show SEM. (D) Representative $D1_{ER}$ recording of DKO cells expressing a control plasmid during store depletion and refilling. Cells were transiently exposed to CPA in Ca^{2+} free medium and 2 mM Ca^{2+} added 3 min later to promote store refilling.

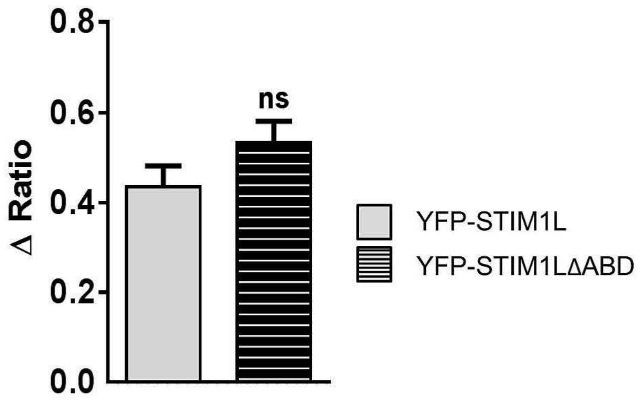

Figure S2 (related to Fig. 2). STIM1LΔABD mediates SOCE.

Quantification of the Ca^{2+} elevations evoked by Ca^{2+} readmission to Tg-treated cells expressing YFP-STIM1L or YFP-STIM1LΔABD (n=23/3/3 and 21/3/3 cells/recordings/transfections; mean±SEM).

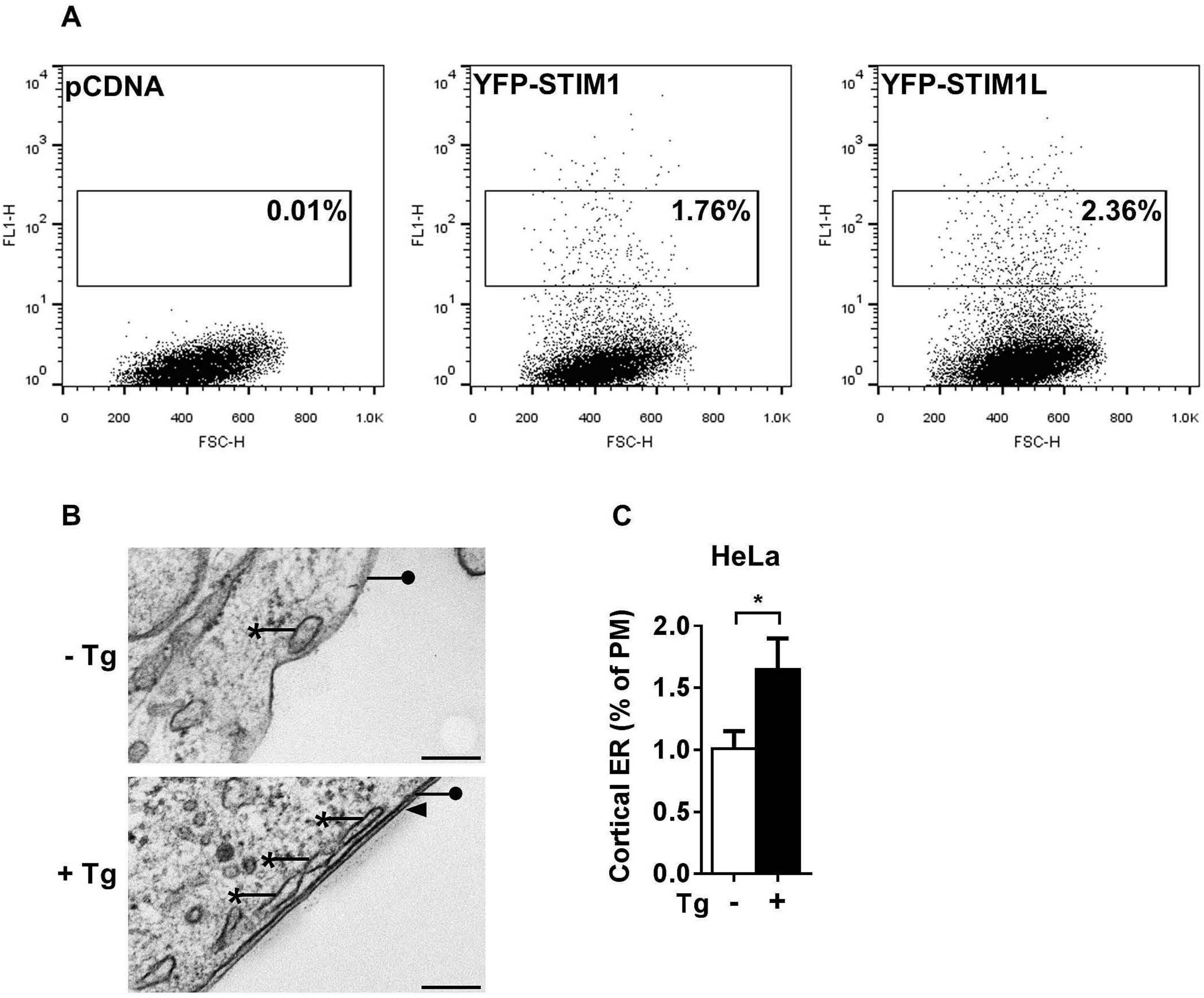

Figure S3 (related to Fig. 3). Fluorescence-activated cell sorting of YFP expressing cells used for EM and ER remodeling in naïve HeLa cells. (A) Representative FACS recordings showing the criteria used to select fluorescent cells for EM. (B) Ultra-structural analysis of HeLa cells expressing a plasmid control (pCDNA) before (top) and 10 min after exposure to 1 µM Tg (bottom), to assess the endogenous ability of cells to remodel their ER. Images show sheets of cER (asterisks) apposed to the PM (closed circle), and arrowhead denotes the dish bottom. Bar: 200 nm. (C) Percentage of PM decorated by cER before and after addition of Tg (n=38-56 for each condition from 2-3 independent experiments).

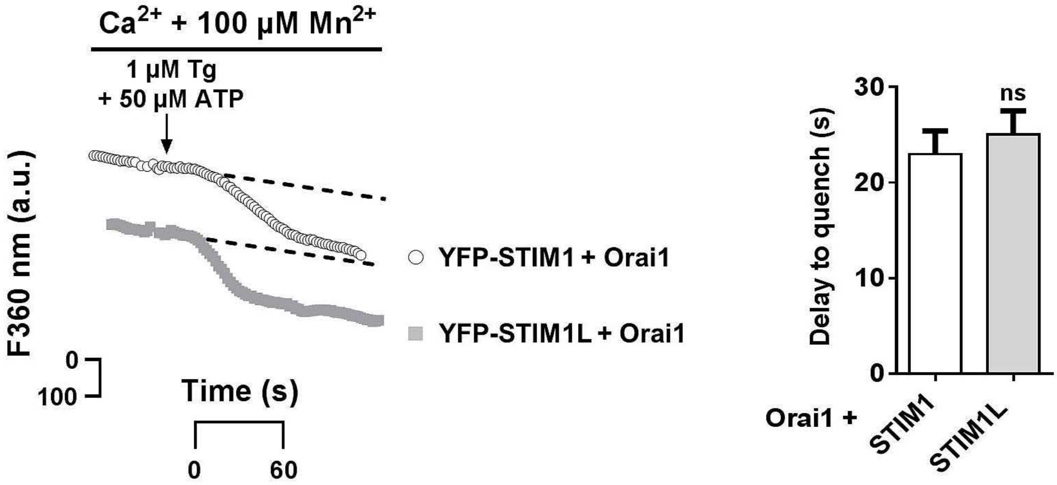

Figure S4 (related to Fig. 4). Kinetics of SOCE activation following fast ER Ca^{2+} depletion. Representative Mn^{2+} quench recordings (left) and quantification (right) of DKO cells co-transfected with Orai1 together with YFP-STIM1 or YFP-STIM1L (n=34/18/4 and 28/10/3 cells/recordings/transfections). Cells were exposed to 100 µM Mn^{2+} prior to 50 µM ATP plus 1 µM Tg addition and fura-2 fluorescence quench was measured at 360 nm.
